# Revisiting Metaplastic Carcinoma of Breast: An Emphasis on the Clinico-pathological and Immunohistochemical Variables Analyzed at a Tertiary Cancer Centre in South India

**DOI:** 10.30699/IJP.2022.541798.2757

**Published:** 2021-08-12

**Authors:** Geetha V Patil Okaly, C Akshatha, N Sandhya, Akina Prakash, M N Suma, Ashwini Nargund, Shankar Anand, C Ramachandra, Libin Babu Cherian

**Affiliations:** 1 *Department of Pathology, Kidwai Memorial Institute of Oncology, Bangalore, India*; 2 *Department of Surgical Oncology, Kidwai Memorial Institute of Oncology, Bangalore, India*

**Keywords:** CK: cytokeratin, EGFR: epidermal growth factor receptor, IBC: Invasive breast carcinoma, IHC: immunohistochemistry, NST: No special type, MC: Metaplastic carcinoma

## Abstract

**Background & Objective:**

Metaplastic carcinoma is a diverse variant of invasive breast carcinomas (IBC) characterized by dedifferentiation of malignant cells towards squamous and/or mesenchymal elements. It accounts for 0.3-1.2% of all IBC. These tumors are typically triple-negative by hormonal profiling with a high proliferation index and a dismal prognosis. Lymph node metastasis is an unusual feature in metaplastic carcinoma.

**Methods:**

The present study analyses 30 cases (26 cases of modified radical mastectomy and 4 cases of lumpectomy) of metaplastic carcinoma over 2018-2020 (3 years). Four oncopathologists reviewed routine histopathologic and immunohistochemical-stained slides. The clinical details were collected from the Medical Records Department of the Cancer Institute.

**Results:**

A total of 20 (66.67%) cases were patients >50 years of age, 21(70%) out of which were diagnosed as invasive carcinoma, grade 3 according to the Nottingham histological score. Five (16.7%) cases presented with lymph node metastasis. While immunohistochemically 28 (93.3%) cases were triple-negativeCK5/6, P63, EGFR, and Ki-67 (more than 40%) positivity was noted in 25 (83.3%) , 26 (86,7%) , 20 (66.7%), and 25 (83.3%) cases, respectively.

**Conclusion:**

Metaplastic carcinoma is characteristically triple-negative breast malignancies (TNBC) exhibiting a high Ki-67 index and a lower rate of lymph node metastasis. CK5/6, p63, and EGFR are pertinent immunohistochemical markers that may aid in diagnosis. However, those markers are non-specific for the disease and morphologic features are always the key to diagnosis of the process.

## Introduction

Metaplastic carcinoma (MC) is a diverse cluster of invasive breast cancers characterized by transforming the neoplastic epithelium towards epithelial and/or stromal-looking elements. MC can affect any anatomical area of the breast. They present at an advanced stage, and their etiology is multifactorial (1). It is triple-negative by hormonal profile, and pathogenesis is associated with late-stage tumor dedifferentiation than arising from basal-type stem cells. TP53 and PIK3CA are the most frequently mutated genes with decreased expression of E-cadherin and increased expression of molecules of epithelial-stromal transition such as SNAIL, TWIST, and SLUG. The tumors present as a well-circumscribed mass or display indistinct irregular borders. Cystic degeneration is not uncommon, especially in MC associated with squamous cell carcinoma (2,3). The variant histological types of MC include the low-grade type of MC, namely adenosquamous carcinoma, fibromatosis like metaplastic carcinoma and the high-grade types, spindle cell carcinoma, squamous cell carcinoma, metaplastic carcinoma with heterologous mesenchymal differentiation and mixed metaplastic carcinoma. The majority of cases express CK5/6, p63, and EGFR. EGFR is commonly amplified in these tumors, and somatic mutation appears to be vanishingly rare. Myoepithelial markers like SMA, CD10, and mapsin are positive in 50-70% of cases. CD34, desmin, and SMMHC are frequently negative. There may be an aberrant expression of beta-catenin. Whole exon and targeting sequencing analysis of MC have demonstrated complex genomic landscape mutations including TP53, RBI, mutations in chromatin remodeling genes ie, *arid1a, kmt2c1,* as well as mutations in *pi3k-akt* pathway genes including *pik3ca, pik3cb, pik3cg, pik3r1, akt1, akt2,* and *akt3* and *ras- raf-mapk* pathway (NF1, KRAS, and NRAS) and WNT pathway (FAT1, CCND3, and CCN6) (4,5) Other histological variants of invasive breast carcinoma include invasive breast carcinoma, NST, tubular carcinoma, cribriform carcinoma, mucinous carcinoma, mucinous cystadenocarcinoma, carcinoma with apocrine features, and micropapillary carcinoma. The various variants of invasive breast carcinoma, NST includes medullary variant, lymphoepithelial variant, glycogen-rich variant, clear cell variant, sebaceous variant, invasive carcinoma with Choriocarcinoma like features, and invasive carcinoma with pleomorphic giant cells.

## Material and Methods

Tissue Blocks and histopathologic slides of thirty MC cases were retrieved from the pathology department archives, and corresponding immunohistochemistry slides were procured for three years, from 2018 to 2020. The slides were reviewed by two experienced oncopathologists. Relevant clinical information was also obtained from the patients and corroborated with the histopathological findings**.**

## Results

Of the modified radical mastectomy, 26 cases and 4 cases of lumpectomy were studied. All cases of MCs were ER, PR, and Her2/neu negative. A total of 20/30 (66.6%) cases were patients more than 50 years of age, with 12 (40%) cases presenting with skin ulceration, satellite nodules, or inflammatory carcinoma. Also, 21 (70%) cases were Nottingham histologic grade 3. Different histopathological variants of MC were streamlined, including 10 (33.3%) cases of adenosquamous carcinoma, 4 (13%) cases of fibromatosis like MC, 5 (16%) cases of spindle cell carcinoma, 7 (23%) of squamous cell carcinoma and 4 (13%) of MC with heterologous elements. In addition, 5 (16.6%) cases presented with lymph nodal metastasis, with one case involving more than six lymph nodes were observed. Of the total cases, 28 (93.3%) were of triple-negative immunophenotype, and 2 (6%) were ER and PR IHC- with Her2/Neu positive, while 25 (83.3%) cases, 26 (86.7%) cases, 20 (66.67%) cases were positive for CK5/6, p63, and EGFR, respectively. Twenty-five (83.3%) cases showed a high proliferation index >40%.

**Fig. 1:**
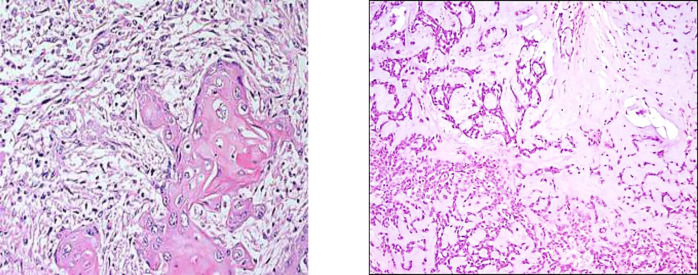
A: photomicrograph showing epithelial elements composed of squamous elements. B: photomicrograph showing mesenchymal elements composed of the chondroid stroma

**Fig. 2:**
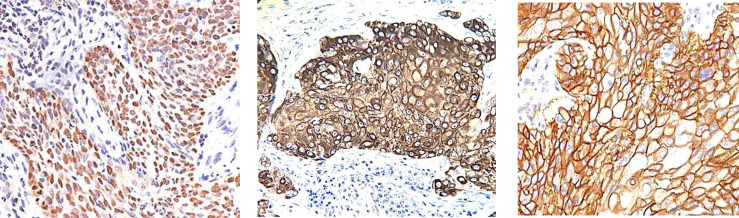
A: p63 diffuse nuclear staining in the squamoid elements. B: CK5/6 diffuse cytoplasmic staining in the squamoid elements. C: EGFR diffuse membrane staining in the squamoid elements

**Fig. 3:**
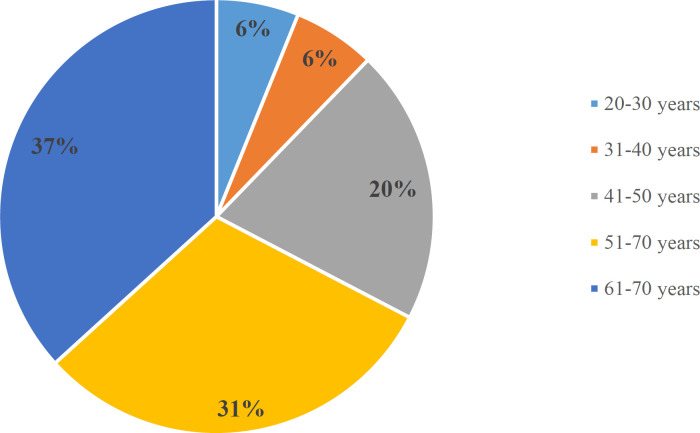
Age distribution in the cases of MC

**Table 1:** Age range of the cases of MC

Age group	Number of cases
20-30 years	**2 (6%)**
31-40 years	**2 (6%)**
41-50 years	**6 (20%)**
51-60 years	**9 (30%)**
61-70 years	**11 (36%)**

**Fig. 4:**
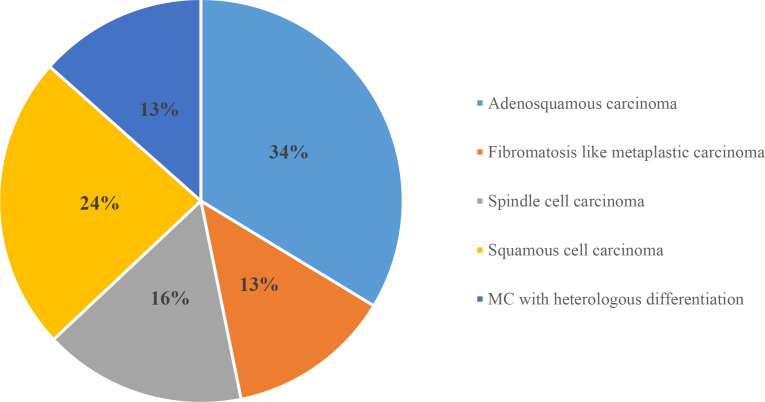
Distribution of histological types of metaplastic carcinoma

**Table 2:** Distribution of the histological types of MC

Histological variants of MC	Number of cases
Adeno-squamous carcinoma	10 (33.3%)
Fibromatosis like metaplastic carcinoma	4 (13%)
Spindle cell carcinoma	5 (16%)
Squamous cell carcinoma	7 (23.3%)
MC with heterologous differentiation	4 (13.3%)

**Table 3:** Stage distribution of the cases of MC

Stage	Number of cases
Stage I	**4 (13%)**
Stage II	**5 (16.7%)**
Stage III	**9 (30%)**
Stage IV	**12 (40%)**

**Table 4:** Distribution of immunohistochemical markers of CK5/6, p63 and EGFR in the cases of MC

	CK5/6		P63	EGFR
Negative	05 (16.7%)		04 (13.3%)	**10 (33.3%)**
Positive	25 (83.3%)		26 (86.7%)	**20 (66.7%)**

**Fig. 5:**
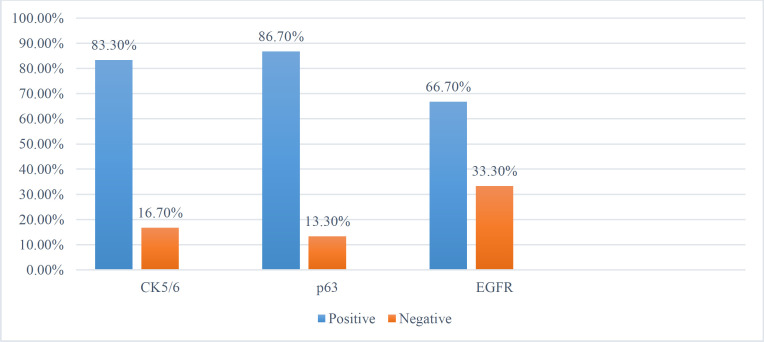
IHC distribution of the cases of metaplastic carcinoma

## Discussion

MC is an infrequent tumor (4% of the malignancies) (6, 7), and constitute only 1.5% of IBC (3). The frequency of MC in our center is 2% among all IBCs. The actual number of cases is not known because it can unusually present as a solitary mass that radiology might miss (8, 9). MC most commonly affects females in the fifth to the sixth decade with a median age of 55 (10,11,12). It usually presents as a palpable firm breast lump, well-circumscribed or occasionally infiltrative borders inflicting any breast quadrant with no quadrant predisposition, unlike the IBC, NST, which has a predisposition to occur in the upper and outer quadrant of the breast. Rarely lesions can involve the skin and chest wall, causing ulceration, peau d'orange appearance, and inflammatory carcinoma in the early course of the disease (13,14). Neither mammograms nor ultrasound of the breast demonstrate specific diagnostic images but show neoplasms most often fairly delineated, without associated microcalcifications. Still, off and on, irregular lesions may be seen (1,10). Breast neoplasms originate from the TDLU, which results in invasive breast carcinoma, finally causing metaplasia of the neoplastic tissue. This differentiation was correlated with the use of IHC (7,15,16,17).

According to the latest edition of the WHO classification, the metaplastic carcinomas are classified into the low grade and the high-grade types, namely low-grade adenosquamous carcinoma, fibromatoses like metaplastic carcinoma, squamous cell carcinoma, spindle cell carcinoma, MC with heterologous elements and unclassified MC. This differentiation is purely based on histopathological grounds, and IHC has no significance. Prognostically spindle cell carcinoma, squamous cell carcinoma, and metaplastic carcinoma with heterologous elements have worrisome prognoses compared to adenosquamous carcinoma and fibromatosis like metaplastic carcinoma. Spindle cell carcinoma and fibromatosis like MC must be differentiated with phyllodes tumor and can become a difficult diagnostic conundrum. Using IHCs such as CK5/6, EGFR and p63 can aid in differentiating from phyllodes tumor (13,14,16,17).

Hormonal profile display ER, PR, her2neu negative in 90% of cases (estrogen receptor-, progesterone receptor-, and human epidermal growth factor receptor 2 (HER2)-negative cases) correlates with our study depicting 93% of cases (3, 11,13).

Lymph node involvement is rarely seen. The incidence of metastases ranges from 5 to 24% (12, 13,17). In invasive breast carcinoma, NST, the incidence is much higher (up to 50%).

Although we could not follow up on these cases, we determined that disease-free survival and overall survival corroborate with the tumor size, in which tumors more than 5 cm have a worse prognosis than smaller tumors. Other factors include histological type, Nottingham histological score, axillary lymph node metastasis, and distant metastasis. Although axillary lymph node involvement is rare, there is an inclination towards pulmonary metastases; so TNM pathological staging system is of little use as a prognostic factor. Most distant metastases occur through the hematogenous route, most frequently affecting the pleura, lungs, liver, and abdominal viscera (2, 8, 12, 21).

Fadwa* et al. *detailed that 85.7% of MC cases expressed luminal breast type of cytokeratins (CK8, CK18 and/or CK19). Out of the five cases (70-75%), three cases were carcinosarcomas, and two cases were SCCs that displayed IHC expression to EGFR. Increased expression of ERBB1 was reported in 80-85% of cases of MBC, with up to 25-38% of cases confirmed by reflux fluorescent in situ hybridization. ERBB1 showed association with squamous or spindle differentiation. Although MBC has been proclaimed to have high levels of ERBB1 upregulation and amplification, they were found to lack ERBB1 activating mutations; therefore, it is obscure whether EGFR tyrosine kinase inhibitors are effective for the management of MBC (31).

Gary M Tse *et al.* emphasized that in MC with epithelial component only, p63 was only expressed in the epithelial squamous cell type but not in the glandular component. Eight of the 10 neoplasms were immunopositive for p63. For the malignancies with spindled type, either singly or in combination with an epithelial component, p63 exhibited expression in 14 of 24 cases. Pure stromal and epithelial types were all immunonegative for p63 IHC staining by immunohistochemistry, thus making p63 IHC staining highly sensitive and specific for confirming metaplastic carcinoma (32).

Five-year overall survival may range from 40 to 68% (8, 13,17), and over 50% of the regional and/or distant metastatic recurrences appear before that time. The management line is modified radical mastectomy, with axillary lymph node dissection and sentinel lymph node biopsy (SLNB) or radical/supra mastectomy, depending on the extent of the tumor dissemination (22,23, 24, 25). MC is usually less amenable to chemotherapy and radiation therapy (27,28,29,30). A variant of MC called matrix-producing metaplastic carcinoma appears to be prognostically better compared to the other variants (18, 19, 26). 

## Conclusion

Metaplastic carcinoma is classically TNBC with a high MIB labeling index and lower lymph node metastasis rate than other invasive breast carcinoma variants. CK5/6, p63, and EGFR are pertinent immunohistochemical markers for MC that may aid in the diagnosis. While these IHC markers are important in distinguishing MC from phyllodes tumors, these markers are non-specific for the disease and morphologic features are always the key for diagnosis. 

## Conflict of Interest

The authors declared no conflict of interest.

## Funding

None.
